# Evaluation of sexual functions and sexual behaviors after penile brachytherapy in men treated for penile carcinoma

**DOI:** 10.1186/2051-4190-24-13

**Published:** 2014-08-28

**Authors:** Patrice Njomnang Soh, Boris Delaunay, Elie Bou Nasr, Martine Delannes, Michel Soulie, Eric Huyghe

**Affiliations:** Department of Andrology and Sexology, Paul Sabatier University, Toulouse University Hospital Paule de Viguier, Toulouse, EA 3694 France; Département d’Urologie CHU Rangueil, 1 av. Jean Poulhès, TSA 50032, 31059 Toulouse Cedex 9, France; Department of Radiotherapy, Institut Universitaire du Cancer, Toulouse, France

**Keywords:** Brachytherapy, Sexual function, Questionnaire, Penile carcinoma, Erection, Curiethérapie, Fonction sexuelle, Questionnaire, Cancer du pénis

## Abstract

**Purpose:**

To assess sexual functions and behaviors of men treated by penile brachytherapy for a cancer of the penis.

**Materials and methods:**

Thirty eight men (19 patients treated by penile brachytherapy for a cancer of the penis and 19 age paired-matched controls) participated in a survey about sexuality. The mean age of patients and controls were 73.2 +/- 11.7 and 70.0 +/- 10.5 years, respectively (NS). Controls were men without penile pathology, without history of cancer and no evidence of cognitive impairment. All agreed to participate in the survey about sexuality using 2 questionnaires : the IIEF questionnaire, which explores 4 domains of sexual functions, namely erection, satisfaction, orgasm and desire, and a questionnaire created using the BASIC IDEA grid, which addresses nine domains: behavior, affect, sensation, self-image, cognition, interpersonal, drugs, expectation and attitude.

**Results:**

Patients had better scores than controls in 3 domains of the IIEF: erection, desire and satisfaction. These results contrasted with the frequency of intercourse and the quality of erection (evaluated through the BASIC IDEA questionnaire) that were not significantly different between the two populations. Patients also had significantly higher frequency of masturbation (p <0.001) lower worry about sexual performance and higher expected satisfaction for future life (p: 0.021) than controls.

**Conclusion:**

Penile brachytherapy is a treatment of cancer of the penis that seems to have a moderated impact on sexual functions since most of sexual scores are not inferior in these patients than in age pair-matched controls.

## Résumé

**Objectifs:**

Évaluer les fonctions et comportements sexuels des hommes traités par curiethérapie pénienne pour un cancer du pénis.

**Materiels et méthodes:**

Trente huit hommes (19 patients traités par curiethérapie pénienne pour un cancer du pénis et de 19 témoins appariés sur l’âge) ont participé à une enquête sur la sexualité par questionnaire. L’âge moyen des patients et des témoins était de 73.2 +/- 11.7 ans et 70.0 +/- 10.5 ans, respectivement. Les témoins étaient des hommes sans pathologie pénienne, sans antécédent de cancer et aucune preuve de troubles cognitifs. Tous les patients ont accepté de participer à l’enquête sur la sexualité qui utilisait 2 questionnaires: le questionnaire IIEF, qui explore 4 domaines de fonctions sexuelles, à savoir érection, satisfaction, orgasme et désir, et un questionnaire créé en utilisant la grille BASIC IDEA, qui aborde 9 domaines: comportement, affect, sensation, image de soi, connaissances, rapports interpersonnels, médicaments, espérance et attitude.

**Résultats:**

les patients ont eu de meilleurs scores que les témoins dans 3 domaines de l’IIEF: érection, désir et satisfaction. Ces résultats contrastaient avec la fréquence des rapports et la qualité de l’érection (évaluée par le questionnaire BASIC IDEA) qui n’étaient pas différents entre les patients et les témoins. Les patients avaient également une fréquence significativement plus élevée de la masturbation (p <0.001) et de l’inquiétude des capacités sexuelles (p: 0.021) que les témoins, ce qui pourrait refléter un effet bénéfique de la masturbation dans cette population.

**Conclusion:**

La curiethérapie pénienne est un traitement du cancer du pénis qui semble avoir un impact modéré sur les fonctions sexuelles, puisque la plupart de scores sexuels chez les patients ne sont pas inférieurs à ceux des témoins appariés sur l’âge.

## Introduction

Penile cancer is a rare disease in Europe and North America, with an incidence between 0.4 and 0.6% in Europe; reaching however 20% in some Asian, African, and South American countries [1–3].

Most tumors of the penis are squamous cell carcinomas and occur most commonly on the glans, prepuce and the coronal sulcus.

Penile brachytherapy (PB) is proposed in small distal lesions of the penis with an objective to preserve sexual quality of life [4]. However, so far, only a few data limited to small descriptive series are available about sexual functions following PB [4–7]. Their results concluded to a moderate impact on sexual functions, but the literature showed several limits: often series included several treatment modalities (surgeries), follow-up was very variable, validated questionnaire were not used in all cases and no study included a control group.

To our knowledge, this study is the first case control study of sexual functions and sexual behaviors in this population.

## Materials and methods

### Patients

Between 1992 and 2009, 35 patients were treated for penile cancer by interstitial Penile Brachytherapy (PB) in the centers of Toulouse (Institut Claudius Regaud, n = 31) and Montpellier (Centre Val D’Aurelle, n = 4).

All patients had a T1 stage well differentiated tumor, with a size <20 mm. None of the patients had palpable lymph nodes on initial physical examination. The treatment consisted of exclusive PB in all cases, with a low dose rate by manual implantation of Iridium-192.

**Figure 1:**
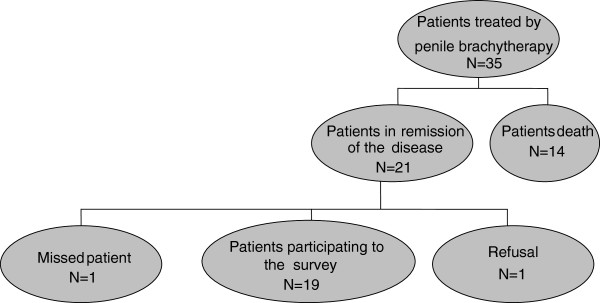
**Flow chart of patients.**

In 2010, we decided to perform a mailed survey about sexuality in men treated by PB without evolutive disease and to compare their results with those of an age-matched 1:1 control group.Figure 1 represents the flow chart in the patients group.

### Constitution of the control group

The age-matched 1:1 control group was recruited in the urology department of the University Hospital of Toulouse in men without penile pathology, without history of cancer and no evidence of cognitive impairment (ie score < 11 on the Orientation-Memory-Concentration Test).

### Modalities of the survey

The study was approved by our institutional research board. An information letter presenting the objectives of the survey was given together with the questionnaire to all the selected patients and controls. Questionnaires were self-administered. Patients were considered as accepting to participate in the survey if they filled out the questionnaire.

### Sexuality questionnaire

We used the French version of the International Index of Erectile Function (IIEF)-15 questionnaire [8] and a questionnaire specially created for the survey following the BASIC IDEA grid [9, 10]. Concerning the latter questionnaire, we paid special attention so that men with or without penile cancer could equally respond to all the questions. The whole questionnaire includes 46 questions and takes about 30 minutes to be completed.

The International Index of Erectile Function is a questionnaire in 15 questions whose answers range between 0 to 4 or 5 per question. It makes it possible to evaluate various aspects of male sexuality in a semi-quantitative manner. Four domains are explored: erection, satisfaction, orgasm and desire. It was developed in the USA by Rosen et al. [8] and since was translated and validated in many languages, including in French. A simplified questionnaire called the five-item International Index of Erectile Function (IIEF-5), comprising the 5 questions of the IIEF-15 with the highest discriminating power to diagnose erectile dysfunction, was extracted [11]. It consists of four questions on erectile function, and one on intercourse satisfaction. The answers are scored on a 5-point scale and the IIEF scores are obtained by calculating the sum of the answers with 5 being the worst possible score and 25 the best [11].

Furthermore, we used the BASIC IDEA grid [9, 10] that addresses nine domains: Behaviour (B), Affect (A), Sensation (S), Imagery (I), Cognition (C), Interpersonal relationship (I), use of Drugs (D), Expectation (E), and Attitude (A).

### Statistical analysis

The software used for the statistical data was Stata^®^ (Stata Corp., College Station, Texas, USA). The chi-square test (*χ*2) or Fisher’s exact (F) were used for comparison of qualitative variables. The student test, Wilcoxon or Kruskal-Wallis tests were used for the comparison of the distributions of quantitative variables. All tests were performed in accordance with their conditions of application with a significance level α = 5%.

## Results

### Characteristics of the population

Thirty eight men (19 patients and 19 controls) participated in the survey between January and September 2010. Characteristics of the population are shown in Table 1. All participants were Caucasians and were retired.

**Table 1:** **Characteristics of the population (=38)**

	Cases (n = 19)	Controls (n = 19)	p
**Age**			
Mean ± SD.	73.2 ± 11.7	70.0 ± 10.5	0.381
Median	76	73	
Min--max	47-89	45-84	
**Diabetes**			
Yes	3	3	1
No	16	16	
**Cardiovascular disease** ^a^			
Yes	12	14	0.485
No	7	5	
**Surgery**			
Orchiectomy	1	1	0.792
Other surgeries (without risk of ED)*	8	6	
No surgery	10	12	
**Marital status**			
Single	0	3	0.071
In couple	19	16	
**Age of partner**			
Mean ± SD.	66.6 ± 14.2	65.4 ± 13.6	0.792
Median	70	68	
Min-max	37-85	37-88	
**Duration of common life (years)**			
Mean ± SD.	38.2 ± 20.4	40.3 ± 16.8	0.731
Median	40	46	
Min-max	4-67	10-65	

The statistical test used for qualitative variables is the chi2 and for quantitative variables, the T Student test. p < 0.05 indicates a significant difference between patients and control.

*NS* no significant.

*Posthectomy (cases = 7; controls = 0), TURP (cases = 1; controls = 3), renal artery stenosis (cases = 0; controls = 1), nephrectomy (cases = 0; controls = 2).

^a^Cardiovascular diseases: valvulopathy, trouble with rhythm or conduction, angina pectoris, cardiac insufficiency, atherosclerosis, myocardial infarction, cerebral vascular infarction, arteritis.

The average age of sexual partner was not statistically different between both groups: 66.6 years (median = 70, range: 37–85) for patients and 65.4 years (median = 68, range: 37–85) for controls.

The average duration of common life was not statistically different: 38.2 years for patients and 40.3 years for controls (Table 1).

### Sexuality data of patients before and after treatment

Before brachytherapy treatment, 17 (89.5%) patients declare they are sexually active, of whom 7 (36.8%) have often intercourse. Fifteen declare they never have erectile dysfunction. Fifteen (78.9%) maintain nocturnal erections. Thirteen (68%) declare they always have ejaculation and 14 (73.7%) maintain orgasms to some extent (Table 2).

**Table 2:** **Pre and post-treatment sexual functions (n = 19 patients)**

Parameters	Classification	Pre-treatment (%)	Post- treatment (%)	P
Sexual intercourse				
	Often	7 (36.9)	4 (21.1)	
	Sometimes	5 (26.3)	2 (10.5)	0.006
	Rarely	5 (26.3)	4 (21.1)	
	Never	2 (10.5)	9 (47.3)	
Erectile dysfunction				
	Often	1 (5.3)	3 (15.8)	
	Sometimes	1 (5.3)	2 (10.5)	0.013
	Rarely	2 (10.5)	6 (31.6)	
	Never	15 (78.9)	8 (42.2)	
Ejaculation				
	Often	13 (68.4)	3 (15.8)	
	Sometimes	3 (15.8)	3 (15.8)	<0.001
	Rarely	2 (10.5)	4 (21.1)	
	Never	1 (5.3)	9 (47.3)	
Orgasm				
	Often	14 (73.7)	3 (15.8)	
	Sometimes	3 (15.7)	3 (15.8)	<0.001
	Rarely	1 (5.3)	4 (21.1)	
	Never	1 (5.3)	9 (47.3)	
Nocturnal erections				
	Often	15 (78.9)	7 (36.9)	
	Sometimes	2 (10.5)	4 (21.1)	0.002
	Rarely	1 (5.3)	4 (21.1)	
	Never	1 (5.3)	4 (21.1)	

The statistical test used is the chi2 test. p < 0.05 indicates a significant difference between patients in pre and post treatment.

After treatment, the proportion of sexually inactive men increases from 10.5% to 47.3%, together with an increase of all sexual dysfunctions : the proportion of men experiencing erectile dysfunction (from rarely to often) increases from 21.1% to 57.8%, and 42% new patients have an absence of ejaculation and orgasm (Table 2).

Globally, all parameters of sexual functions in Table 2 showed a significant difference between pre and post-treatment of patients.

Among the patients who continued to have sexual intercourse, 8 (80%) maintained orgasms. Patients who felt that PB had little or no changes in their sexuality had an IIEF-5 score (p = 0.016), IIEF-15 (p = 0.003) and a frequency of sexual intercourse (p = 0.026) significantly higher. We found no significant correlation among the sexuality items and the parameters of PB (dose, dose rate, number of needles, active length) and the tumor size (data not shown). The level of sexual desire was correlated with the frequency of sexual intercourse before (p: 0.0498) and after treatment (p = 0.0009), and to the satisfaction of sexual intercourse (p = 0.00001). The age of the patients and their partners were inversely correlated with the level of sexual desire (p = 0.0093 and p = 0.0113).

### Comparison of sexual functioning between patients and control

Three questions rank higher in patients than controls: the frequency of masturbation with 79% of patients declaring that they often proactice it vs 16% in controls (p <0.001), absence of worry about sexual performance (p: 0.021) and expected satisfaction for future sexual life (p: 0.021) (Table 3). We also noted significant higher scores in patients than controls concerning several domains of IIEF5 (erectile function, sexual desire and overall satisfaction) (Table 4). These results contrasted with data of the BASIC IDEA questionnaire regarding the frequency and the quality of erection, which were not significantly different between patients and controls.

**Table 3:** **Responses to the questionnaire on sexuality (n = 38)**

	Cases (n = 19)	Controls (n = 19)	P
**Frequency of sexual intercourse**			
never	9 (47)	12 (63)	0.580
rarely	4 (21)	2 (11)	
sometimes	2 (11)	3 (15)	
often	4 (21)	2 (11)	
**Frequency of erection**			
never	2 (11)	6 (32)	0.075
rarely	4 (21)	8 (42)	
sometimes	5 (26)	2 (10)	
often	8 (42)	3 (16)	
**Frequency of nocturnal erection**			
never	4 (21)	9 (47)	0.231
rarely	4 (21)	5 (26)	
sometimes	4 (21)	2 (11)	
often	7 (37)	3 (16)	
**Quality of erection**			
soft	4 (24)	4 (31)	0.892
slightly hard	3 (18)	3 (23)	
almost hard	6 (35)	3 (23)	
hard	4 (24)	3 (23)	
**Frequency of masturbation**			
never	0 (0)	0 (0)	<0.001
rarely	0 (0)	4 (21)	
sometimes	4 (21)	12 (63)	
often	15 (79)	3 (16)	
**Worry about sexual performance**			
never	17 (89)	11 (58)	0.021
rarely	2 (11)	1 (5)	
sometimes	0 (0)	3 (16)	
often	0 (0)	4 (21)	
**Discomfort due to penile length**			
absent	16 (84)	15 (79)	0.258
slight	3 (16)	1 (5)	
moderate	0 (0)	2 (11)	
strong	0 (0)	1 (5)	
**Discomfort due to penile appearance**			
absent	14 (74)	15 (79)	0.667
slight	3 (16)	2 (11)	
moderate	2 (10)	1 (5)	
strong	0 (0)	1 (5)	
**Feeling as a true man**			
never	0 (0)	0 (0)	0.107
rarely	0 (0)	3 (16)	
sometimes	0 (0)	1 (5)	
often	19 (19)	15 (79)	
**Frequency of fantasy**			
never	9 (47)	3 (16)	0.091
rarely	6 (32)	7 (37)	
sometimes	4 (21)	6 (31)	
often	0 (0)	3 (16)	
**Stopping intercourse**			
our decision	8 (89)	5 (42)	0.146
the decision of my partner	1 (11)	3 (25)	
my decision	0 (0)	1 (8)	
not answered	0 (0)	3 (25)	
**Partner ‘s trouble during intercourse**			
yes	2 (10)	1 (5)	0.266
no	10 (53)	6 (32)	
not answered	7 (37)	12 (63)	
**Importance of sexuality for the partner**			
Very important	4 (21)	1 (6)	0.100
important	7 (37)	8 (50)	
Merely important	7 (37)	2 (12.5)	
Not important	1 (5)	2 (12.5)	
not answered	0 (0)	3 (19)	
**Partner’s coping with sexual troubles**			
Accepts and understands	14 (74)	7 (47)	0.294
Wants me to recover my potency	1 (5)	0 (0)	
Is disappointed	1 (5)	1 (7)	
Does not mind	3 (16)	6 (40)	
No advice	0 (0)	1 (7)	
**Drug consumption to improve potency**			
often	1 (5)	1 (5)	0.890
rarely	2 (11)	3 (16)	
never	16 (84)	15 (79)	
**Expected satisfaction for future Sexual life**			
High satisfaction	12 (63)	8 (42)	0.021
moderate satisfaction	5 (26)	1 (7)	
Mild satisfaction	2 (11)	5 (26)	
No advice	0 (0)	5 (26)	

The statistical test used for qualitative variables is the chi2. p < 0.05 indicates a significant difference between patients and control.

**Table 4:** **Comparison of IIEF scores between patients having intercourses (n = 17) and controls**

	Cases					Controls					
Variable	Mean	SD	Median	Min	Max	Mean	SD	Median	Min	Max	P
Erectile function	14,3	13,0	5	1	30	9,5	11,6	2	0	28	0.03
Orgasmic function	4,5	5,0	0	0	10	4,4	4,3	2	0	10	0.7
Sexual desire	5,9	2,3	6	2	10	4,1	2,2	5	0	8	0.027
Intercourse satisfaction	5,4	5,8	2	0	15	4,0	5,2	0	0	12	0.3
overall satisfaction	8,2	2,4	10	4	10	5,6	3,2	8	0	8	0.004
IIEF 15	38.3	26.8	21	8	74	27.5	23.9	14	5	62	0.039
IIEF 5	12.0	10.6	5	1	25	7.8	9.8	1	0	24	0.018

The statistical test used for quantitative variables is the wilcoxon test. p < 0.05 indicates a significant difference between patients and control.

All other questions of the BASIC IDEA questionnaire (frequency of sexual intercourse, discomfort due to penile length, discomfort due to penile appearance, feeling as a true man, frequency of fantasy, stopping intercourse, partner ‘s trouble during intercourse, importance of sexuality for the partner, partner’s coping with sexual troubles, drug consumption to improve potency) were not different between patients and controls.

### Parameters of patients after PB

#### Aspect of the penis after treatment and sensitivity

**Figure 2:**
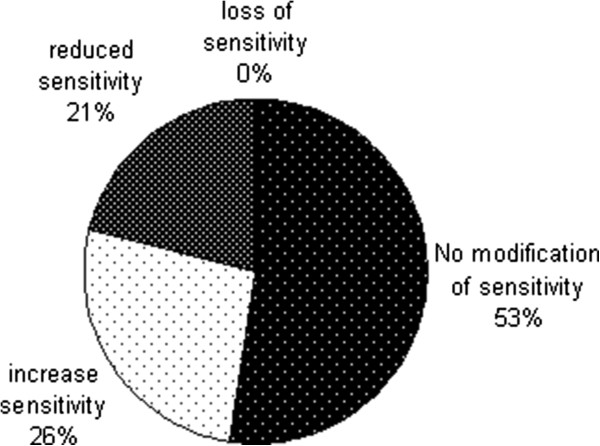
**Glans sensitivity after penile brachytherapy.**

Three (15.7%) patients were concerned by the size of their penis. Ten men (52.6%) observed modifications in glans sensitivity (Figure 2). The hypersensitivity was a little more frequent than the loss of sensitivity. These two situations are both susceptible to perturb sexual life. Changes in sensitivity of the glans, the discomfort or the appearance of the penis, pain and ulceration were not significantly related to changes in sexuality. No patient felt a loss of manliness or were worried about sexual performance.

#### Sexual fantasy

**Figure 3:**
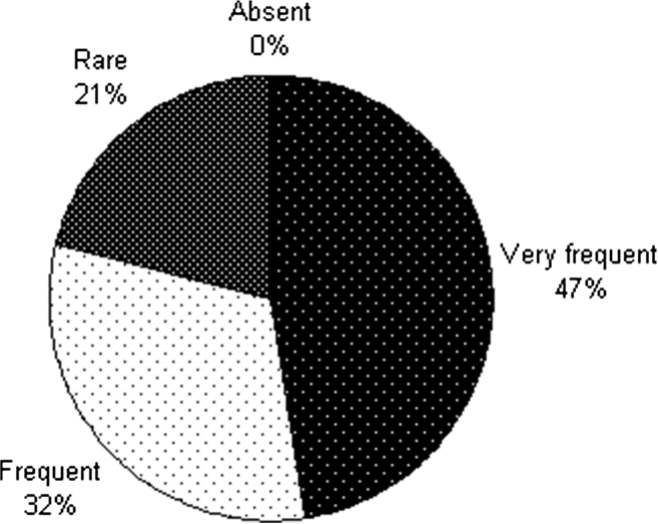
**Sexual fantasy after penile brachytherapy.**

All men (100%) said they had “often” to “very often” erotic fantasies (Figure 3).

#### Sexual desire

**Figure 4:**
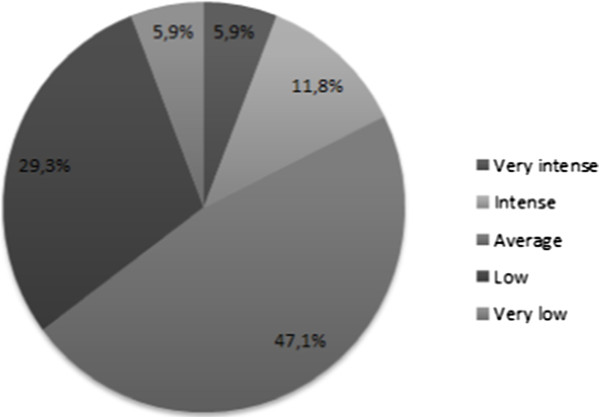
**Sexual desire intensity after penile brachytherapy.**

Only 2 (10.5%) patients declared a loss of desire. The intensity of desire was described as “intense” to “very low” (Figure 4). Seven (35.2%) had a low or very low desire.

#### Partner and couple relationship

**Figure 5:**
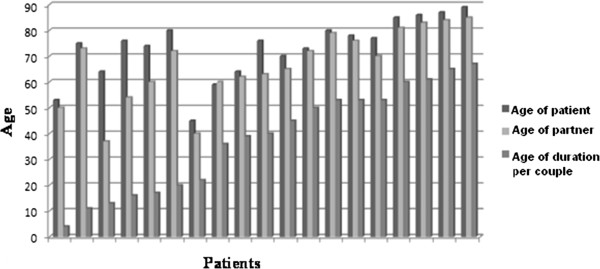
**Women and men age per couple duration.**

**Figure 6:**
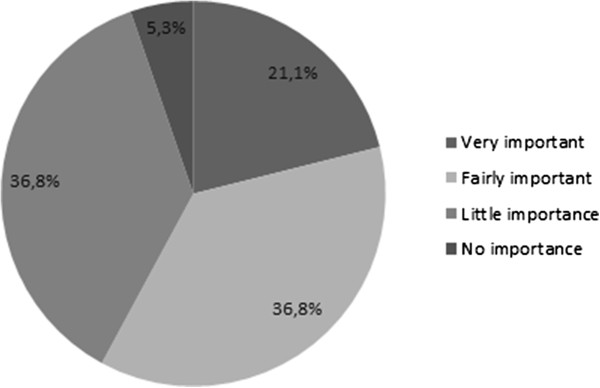
**Importance of sexuality for the partner.**

Only one patient had an older partner. The median difference of age between the patients and their partners was of 4 years; whereas 4 couples had a difference of age exceeding 10 years (ranging from 13 to 27) (Figure 5). The average duration of cohabitation was 38.2 years (median = 40 years, min = 4 years, max = 67 years).Eleven (57.9%) patients felt that sexuality was “important” or “very important” for their partner, and only one patient (5.3%) declared that sexuality was not important at all for his partner (Figure 6).

**Figure 7:**
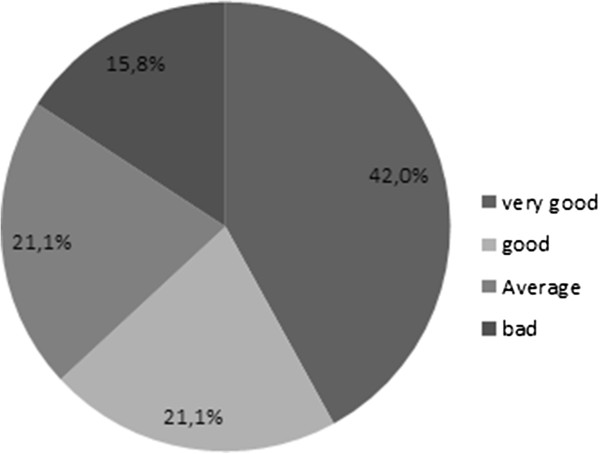
**Communication about sexuality with partner.**

Fourteen patients (73.6%) declared that their partner “accepted and understood” their sexual dysfunction, one (5.3%) patient said that she “was disappointed”, three (15.8%) that she “didn’t express any opinion concerning sexuality” and finally one (5.3%) patient declared that she “wished I recovered my sexual performance”.A total of 12 (63.1%) patients considered that together with their partner, they had a “good” (n = 4) or “very good” (n = 8) communication about sexuality. Only 3 (15.8%) patients declared they never discussed about sexuality with their partner (Figure 7).

#### Overall sexual satisfaction

More than 1 in 2 patients declared being “very satisfied” of his sexual life (n = 11; 57.9%), and 8 (42.1%) reported an “average satisfaction”. No patient was really disappointed of his sexuality after treatment.

## Discussion

The purpose of this study was to compare sexual functions and psychosexual quality of life in men treated by PB for penile cancer with age-matched controls to further understand the impact of treatment in this population. So far, little information was provided in the literature on the effects of PB on sexual behavior. All the studies evoked the persistence of sexuality after PB [6, 7, 12], without providing detailed analysis of sexual functions and behaviors. By analyzing a series of 51 patients treated between 1971 and 1989, Delannes et al. concluded that PB does not seem to impact sexual functions [5]. However, they did not use a questionnaire and there was no control group. The current study first assesses couple relationship and communication about sexuality, sexual behaviors, affect and fantasy in this population, compared with controls.

We found that a relatively high proportion of patients have erectile dysfunction after penile brachytherapy (using the IIEF as well as the BASIC IDEA questionnaires). However, our results show that erectile function IIEF subscores are better in patients after treatment than in controls.

This difference may be subtle, as illustrated by the absence of difference between patients and controls concerning frequency of erection, frequency of nocturnal erection and quality of erection using the BASIC IDEA questionnaire.

Our results are also important in that we found that both PB patients and age-matched controls similarly experienced relatively few problems with sexual functioning and sexual behavior.

In a recent review of sexual functioning and mood in men treated for penile cancer, Maddineni et al. [13]. concluded that all penile cancer treatments result in negative effects on well-being in up to 40%, with psychiatric symptoms in approximatively 50%, and up to two thirds of patients reporting a reduction in sexual function. However, this review has several limitations: First, a majority of studies only used the International Index of Erectile Function Questionnaire (IIEF) for the evaluation of sexual functions; this questionnaire is centered on erectile function and clearly limited to analysis of sexuality of men who have intercourse. Another limitation of this review is the lack of detailed information about cancer treatment modality. Among treatments of localized penile cancer, several surgical treatments, namely partial penectomy, glansectomy, and glans resurfacing, may lead to unsightly scarring and shortening of penis that may have an impact on both sexual function, as well as self-image and self-esteem. Several anatomic structures involved in erectile function may be affected by PB, notably intrinsic cavernosal erectile tissue and nerves, this by an increase radiation risk.

In our study, all patients declared that their manliness had not been altered by the treatment and there was no difference between men treated by PB and age-matched controls. The study shows that more than three quarters of patients declare they do not have any discomfort due to penile length or penile appearance. This is probably an important determinant of their feeling as a true man. We also observe that communication about sexuality in their couple, importance of sexuality for the partner, partner’s coping with sexual troubles were significantly better in the patients than in controls and that patients had more fantasy than controls. No doubt that all these factors enable that to minor the impact of treatment on glans sensitivity and erectile function, and strengthen their self-esteem and motivation.

Maddineni et al. [13] found a greater impact on sexual functions of partial amputation of the penis, with an absence of sexual function in 36 - 67% of patients. In a series of 17 patients treated with partial (n = 11) or total (n = 4) amputation of the penis, Ficarra et al. [14] found that emotional and mood disorders were common in this population, with 35% of patients experiencing “problems in social life,” 29.5% anxiety and 6% depression. The feeling of loss of manliness and the inability to penetrate is likely to cause emotional stress, and we can presume that many patients treated by total or partial amputation of the penis feel it to varying degrees.

The study showed a higher frequency of masturbation in patients than controls. The question of masturbation as an indication of better psychological and physiological health should be more thoroughly explored. Contrarily to our study, Gerressu et al. [15] observed that men who masturbate have more sexual problems. Moreover, Brody and Costa [16] showed that penile-vaginal intercourse frequency, rather than other sexual activities, was associated with sexual satisfaction, health, and well-being. The authors also found an inverse association between satisfaction and masturbation. Exploring the reasons why men and women masturbate, Das et al. [17] suggest that masturbation is often more than a simply compensatory behavior for regular partnered sex, and that masturbatory patterns are heavily influenced by early sexualization, and low socioeconomic development. The authors conclude that a complex model is needed to comprehend masturbatory practice.

At least, our results show that in such an elderly population, good sexual satisfaction does not implicate necessarily intercourse. In the future, it would be necessary to take into account the multiple representation of the sexuality during exploration of PB treatment on sexual function and sexual behavior, without limiting itself to the penetration capacity.

### Limitations

Sexuality is an area highly dependent on socio-cultural factors; therefore it may be difficult to extrapolate these data to other cultures. In addition, because of the low incidence of this disease in Europe, the size of our study population was relatively small, which limits our ability to achieve a detailed analysis, including subgroups (young males, gay, low socioeconomic status, etc.).

Regarding the methodology, although we have chosen the form of self-administered questionnaire, followed by an interview so the patients are not influenced in their responses and that misunderstanding of the questions is limited, we cannot rule out the subjectivity of responses. In addition, the use of the IIEF in this population is quite questionable because it is a poor score in a population with few penetrating sexual reports. For this reason, we have completed the inquiry by a questionnaire specifically designed for the study. Our results can often be confusing, as in the contradictory results between our questionnaire and IIEF score (Tables 3 and 4 respectively). However, the conclusions drawn from this second questionnaire must be taken with caution due to the absence of validation of our questionnaire. For all these reasons, even if this study gives us the first comprehensive case–control analysis of sexual functions and behaviors in men treated by PB, it should be considered as a pilot study requiring further analysis before that definite conclusions can be drawn.

Finally, a major limitation of the study is the fact that control population had a worse sexuality than patients, possibly due to comorbidities. This should be clarified in future studies.

## Conclusion

Following PB, most of patients report that treatment has little effect on their sexuality compared with control population. More than half of patients remain sexually active after treatment and almost all continue to have erections even if the quality may be deteriorated. There is little damage to body image and sense of manliness. This information may play a key role in the choice of penile cancer treatment leading to the maintenance of a good sexual life.
